# The brain-body disconnect: A somatic sensory basis for trauma-related disorders

**DOI:** 10.3389/fnins.2022.1015749

**Published:** 2022-11-21

**Authors:** Breanne E. Kearney, Ruth A. Lanius

**Affiliations:** ^1^Department of Neuroscience, Schulich School of Medicine and Dentistry, Western University, London, ON, Canada; ^2^Department of Psychiatry, Schulich School of Medicine and Dentistry, Western University, London, ON, Canada

**Keywords:** vestibular, somatosensory, attachment, sensory processing, trauma, post-traumatic stress disorder (PTSD), sense of self, embodiment

## Abstract

Although the manifestation of trauma in the body is a phenomenon well-endorsed by clinicians and traumatized individuals, the neurobiological underpinnings of this manifestation remain unclear. The notion of somatic sensory processing, which encompasses vestibular and somatosensory processing and relates to the sensory systems concerned with how the physical body exists in and relates to physical space, is introduced as a major contributor to overall regulatory, social-emotional, and self-referential functioning. From a phylogenetically and ontogenetically informed perspective, trauma-related symptomology is conceptualized to be grounded in brainstem-level somatic sensory processing dysfunction and its cascading influences on physiological arousal modulation, affect regulation, and higher-order capacities. Lastly, we introduce a novel hierarchical model bridging somatic sensory processes with limbic and neocortical mechanisms regulating an individual’s emotional experience and sense of a relational, agentive self. This model provides a working framework for the neurobiologically informed assessment and treatment of trauma-related conditions from a somatic sensory processing perspective.

## Introduction

Our physical body is the home within which our brain resides, and as such it provides an anchor to who we are and how we move about in the world. The body is connected to the brain via afferent and efferent tracts, or bundles of nerve fibers, which allow swift and efficient integration of sensation, emotion, cognition, and action. Elementally, an organism receives sensory input, contextualizes it with emotional and cognitive information, and effects an appropriate response. As such, sensory *processing* refers the capacity to register, organize, and modulate incoming sensory information from the internal or external milieu, where it is then *integrated* with sensory input from other modalities and utilized to guide a goal-oriented behavioral response (Gilbert and Sigman, 2007; Miller et al., 2007; Alais et al., 2010; Atick, 2011). Here, we con sider how alterations in the neural pathways crucial for the processing and integration of somatic, or body-based, sensations may be off-balance due to severe or chronic traumatization, with the diagnoses of post-traumatic stress disorder (PTSD) and its dissociative subtype (PTSD + DS) as well-researched examples. Alterations to multisensory processing have been shown to mediate higher-order cognition (Alais et al., 2010; Wallace et al., 2020), emotion (Klasen et al., 2012), social capacities (Baranek et al., 2018; Stevenson et al., 2018; Thye et al., 2018), and sense of self (Panksepp and Northoff, 2009; Tsakiris, 2017), which are globally impacted in trauma-related disorders.

An altered neural defense circuitry leading to persistent sensory and emotional overwhelm in response to stimuli is evident in trauma-related disorders. The symptomology of PTSD and PTSD + DS indicates maladaptive responsivity to incoming information, including physiological arousal dysregulation, impulsivity, diminished sense of agency, altered sense of time, and social difficulties or isolation (American Psychiatric Association [APA], 2013; Cox et al., 2014). PTSD is diagnosed after a traumatic event which elicited feelings of terror and/or a threat to one’s or another’s life. Symptoms include flashbacks or dissociative re-experiencing, hypervigilance, increased startle responses, behavioral avoidance of potential triggers, and pervasive negative cognitions (American Psychiatric Association [APA], 2013). Additionally, PTSD + DS occurs in up to 44% of individuals with PTSD and is characterized by additional symptoms of derealization and depersonalization (Lanius et al., 2010b; Steuwe et al., 2012; Wolf et al., 2012b; Stein et al., 2013; Armour et al., 2014; Blevins et al., 2014; for a review, see Hansen et al., 2017; White et al., 2022). In derealization, an individual feels as though their external environment is slowed down, foggy, dream-like, or otherwise unreal, while in depersonalization the individual feels as if they are detached from or even floating above their physical body. PTSD + DS is highly associated with early life traumatization, where dissociation acts as an adaptive response to inescapable threat (Nijenhuis et al., 1998a; Chu et al., 1999; Spiegel et al., 2011; Wolf et al., 2012a; Stein et al., 2013; Lowenstein, 2018). Here, pathological dissociation may be an “internal mechanism by which terrorized people are silenced” (Herman, 1992, p. 239), with adaptive neurobiological mechanisms in place for suppressing the sensory and emotional overwhelm of chronic trauma. During a traumatic experience (peritraumatically) negatively valenced sensory input overwhelms lower-level processing regions within the brainstem and midbrain. Peritraumatically, individuals feel unsafe, under threat, out of control and prevented from getting their (or another’s) survival needs met; trauma-related disorders arise when these perceptions persist post-traumatically. For those with a history of chronic emotional and/or physical neglect, lower-level brain regions are starved for the type of sensory input inherent in positively valenced and safe physical and social interactions. Mammalian survival needs include attuned caregiving during neurodevelopment and reciprocal relationships in adulthood for collective safety, survival, and continuation of the species. Therefore, emotional neglect is traumatizing as it engenders a perpetual fear that survival needs will not be met.

Post-traumatic stress disorder (PTSD) has been broadly associated with sensory modulation difficulties, manifested as either hyper- or hypo-responsivity to sensory input (Shalev et al., 2000; Engel-Yeger et al., 2013; Devine et al., 2020; Yochman and Pat-Horenczyk, 2020; Joseph et al., 2021). Sensory hyper-responsivity is most evident in PTSD and characterized by lower thresholds for registering and thus alerting to incoming sensory information even in the absence of threat, resulting in frequent states of overwhelm. A persistently heightened arousal state due to facilitated threat detection circuitry may explain the enhanced detection of innocuous stimuli as threatening (Harricharan et al., 2021). Alternatively, those with dissociative symptoms experience states of sensory hypo-sensitivity including analgesia/anesthesia (Nijenhuis et al., 1998b), suggesting heightened neurological thresholds for sensory registration. Depersonalization corresponds with somatic sensory hypo-responsivity, while derealization suggests exteroceptive sensory hypo-responsivity. These lowered and heightened thresholds may be linked with under- and over-modulation, respectively, of raw sensory and emotional input from subcortical regions. In PTSD, subcortical regions drive cortical overload, while in PTSD + DS cortical regions over-modulate lower regions resulting in bodily and emotional detachment (Lanius et al., 2010b). Together, traumatogenic conditions may be a manifestation of disrupted subcortico-cortical or *vertical* integration, where the bipartisan modulation between lower and higher regions is off-balance, particularly within the midline neural circuitry poised to engender a primordial sense of bodily and affective self as a coherent and stable entity in relation to its environment (Panksepp and Northoff, 2009). This alteration to vertical integration has a cascading impact on thalamo-cortical and cortico-cortical *horizontal* integration of cortical brain structures. Vertical and horizontal integration relates to neural synchrony, in that lower and higher, as well as medial and lateral, reaches of the brain may be structurally sound yet lacking in fluid communication (Llinás, 1970).

Here, we introduce a neurobiologically informed perspective from which to view trauma-based conditions considering how sensory processing contributes to an organism’s ability to regulate physiological arousal, emotions, and actions (Harricharan et al., 2021). Specifically, we focus on the importance of the *somatic* (vestibular and somatosensory) senses given their direct relevance to the physical body, their positions of phylogenetic and ontogenetic primacy, and the major roles they play in attenuating and orchestrating our multisensory experience in the present moment. Somatic sensory processing is hypothesized to give rise to adequate and efficient processing of interoceptive and exteroceptive (visual, auditory, olfactory, gustatory) sensory information. The overall aim of this review is to offer a transdisciplinary, neuroscientifically informed perspective of how somatic sensory processing contributes to trauma-related symptomology. We review (1) an overview of the vestibular and somatosensory systems; (2) phylogenetic and ontogenetic development of the somatic sensory systems; (3) the neurobiology of sensory processing as mediated by the periaqueductal gray; (4) trauma as an assault on the senses; (5) somatic sensory contributions to the sense of self after trauma; and (6) what neuroscience can teach us about connecting somatic sensory processing and trauma-related disorders.

## Overview of the somatic sensory systems: Vestibular and somatosensory

### The vestibular system

The vestibular system, comprised of angular motion-detecting semicircular canals and linear motion-detecting otolith organs in the inner ear, informs us of three-dimensional head acceleration as a function of the linear pull of gravity (Lopez and Blanke, 2011; Holstein, 2012; Jamon, 2014). It is a sensory system that never “sleeps” – gravity is an omnipresent and unchanging force which perpetually stimulates the vestibular system, impacting everyday movements in relation to the physical and social world around us. As a constant, gravity is predictable and something we come to subconsciously understand during our formative years. As such, developing a reference to gravity is critical in orienting our bodies in space, navigating through the environment, maintaining an upright and vertical orientation as bipeds, and sensing the passage of time as a function of terrestrial motion (Brandt et al., 1994; Lacquaniti et al., 2015; Ferrè and Haggard, 2016). The vestibular system’s workings remain subconscious until we unexpectedly trip or accelerate, increasing autonomic arousal via the vestibular nuclei’s descending projections modulating vestibulo-sympathetic reflexive influences on breathing, heart rate, and blood flow (Hernandez and Das, 2020) and their connections with the reticular activating system (RAS; Peterson et al., 1975). Anyone who has unexpectedly lost balance can attest to the quickened heart rate, dropped sensation in the gut, and gasping that ensue, procuring our immediate attention. These autonomic and motoric responses modulate arousal and maintain our physical safety prior to reaching awareness. Vestibular processing, therefore, is inextricable with a sense of security, grounding, and safety.

**FIGURE 1:**
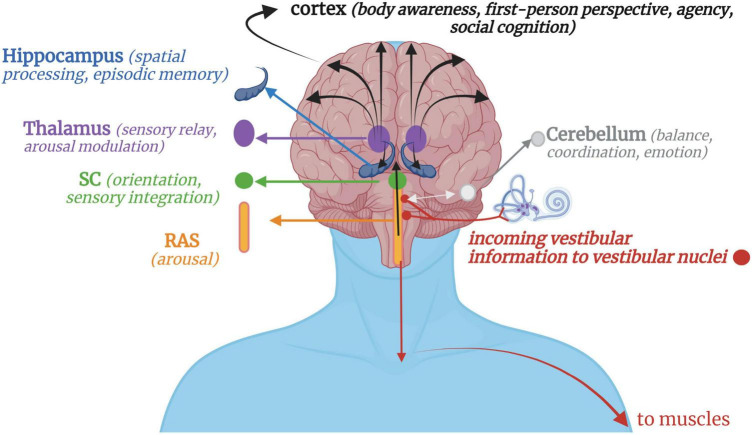
A simplified schematic of vestibular projections throughout the nervous system. Afferent input from gravitational forces arrives at the vestibular nuclei, which integrate with somatosensory input via the cerebellum, reticular formation, and spinal afferent tracts. Downward vestibular efferent projections send signals for muscular extension and postural control. Reciprocal connections with the cerebellum, mainly its flocculonodular lobe, allow for feedback-feedforward mechanisms of motor coordination and fluency, with the cerebellum projecting information back to the vestibular nuclei or directly up to the cortex. Ascending vestibular projections integrate with the RAS and SC for arousal modulation and orienting responses before continuing on to higher cortical structures via the thalamus. Vestibular projections to the cortex then contribute to higher-order cognitive processes such as a sense of agency, first-person perspective, social cognition, and bodily self-consciousness. RAS, Reticular activating system; SC, Superior colliculi.

Vestibular stimulation occurs when crystals and fluid are displaced in the otoliths and semicircular canals, respectively, sending signals to the vestibular nuclei via the pontomedullary junction (Carmona et al., 2009; Jamon, 2014) or the flocculonodular lobes of the cerebellum via the inferior cerebellar peduncle (Highstein and Holstein, 2006). Proprioceptive input from the head, neck and trunk integrates with vestibular input at the vestibular nuclei, thalamus, and cerebellum, contextualizing the motion as self- or other-initiated (Deecke et al., 1977; Gdowski and McCrea, 2000; Luan et al., 2013). Neurons from the vestibular nuclei project down the lateral vestibulospinal tract to influence muscle tone and postural control (Yoo and Mihaila, 2020), down to innervate vestibulo-sympathetic reflexes of the heart and lungs (Hernandez and Das, 2020), across to the flocculonodular lobe in the cerebellum for balance, coordination, and emotion processing (Britton and Arshad, 2019), and up to the inferior and superior colliculi of the midbrain and multisensory integrative areas of the cortex to contribute toward body awareness, spatial processing/memory, arousal modulation, first-person perspective, and social cognition (Ferrè and Haggard, 2020; Rabellino et al., 2022) (Figure 1).

Originally thought as solely involved in balance and oculomotor processes, vestibular projections are now known to be multifaceted in their influences given their widespread innervation of the brain and body (Carmona et al., 2009; Lopez and Blanke, 2011; Ferrè and Haggard, 2016) and their multisensory nature (Raiser et al., 2020; Huber et al., 2021). Based on anecdotal observations, meditative employment of rocking by Buddhist monks, whirling by Sufi semazen-artists, and mindful movements by yogic practitioners speaks to the universal and profound impact vestibular stimulation has on quieting the mind’s chatter and supporting a state of presence and tranquility. Functionally, vestibular input has been shown to be involved in motor control (Nandi and Luxon, 2008; Takakusaki, 2017), autonomic arousal regulation of respiration and blood pressure (Balaban and Porter, 1998; Biaggioni et al., 1998; Furman et al., 1998; Yates and Miller, 1998; Murakami et al., 2002), emotion regulation (Balaban and Thayer, 2001), social cognition (Mast et al., 2014; Deroualle et al., 2015), and maintenance of a coherent representation of the body (Mast et al., 2014; Ferrè and Haggard, 2016). Unlike other sensory systems which have a primary thalamic relay and cortical processing area, no focal processing region dedicated solely to the vestibular system has been defined (Hitier et al., 2014; Wijesinghe et al., 2015). A “vestibular cortex” has been delineated in the primate and other mammals (Akbarian et al., 1992; Guldin et al., 1992; for a review, see Guldin and Grüsser, 1998) but remains to be definitively mapped in the human brain largely due to the confounds presented by its integration with other sensory stimuli (zu Eulenburg et al., 2012; Frank and Greenlee, 2018). The most consistent and robust findings of vestibular-cortical projections have involved the insula and the surrounding parietal operculum, temporo-parietal junction (TPJ), superior temporal gyrus, somatosensory cortex, and mid-cingulate cortex (Guldin and Grüsser, 1998; Lopez and Blanke, 2011; Frank and Greenlee, 2018), the largest hubs being the parietal operculum (OP2) (zu Eulenburg et al., 2012; Huber et al., 2021; Ibitoye et al., 2022) and TPJ (Bottini et al., 1994, 2001; Lopez and Blanke, 2011). These multimodal processing regions are inclusive of auditory, visual, and somatosensory input, suggesting a multisensory binding role played by the vestibular system (Pfeiffer et al., 2013; Shayman et al., 2018). Multisensory binding then gives rise to a unified multisensory experience underlying self-representation and bodily self-awareness (Ferrè et al., 2014). This has been evidenced by lengthened multisensory temporal binding windows in individuals with vestibular hypofunction, such that multisensory inputs take longer for transmission and processing resulting in disjointed arrivals at multisensory integrative brain regions (Shayman et al., 2018).

Vestibular input purportedly modulates and manages sensory integrative processes by balancing sensory signals in response to internal and external environmental demands (Bense et al., 2001; Ferrè and Haggard, 2016). This modulatory role is crucial in multisensory integration, where the balancing of interoceptive and exteroceptive sensory input is critical in creating and maintaining our perception of reality while guiding adaptive and purposeful behavior (Harricharan et al., 2021). Vestibular stimulation has a modulating effect on proprioceptive (Gallagher et al., 2021), tactile, nociceptive (McGeoch et al., 2009; Ferrè et al., 2013), and visual-proprioceptive cues (Ponzo et al., 2018). Further, optogenetic stimulation of the medial vestibular nuclei results in enhanced cortical activations in response to visual and auditory stimuli (Leong et al., 2019), while caloric vestibular stimulation has an analgesic effect (Ferrè and Haggard, 2015) in healthy individuals. Here, vestibular input adjusts perceptual experience in relation to an ever-changing body-environment dynamic, thereby contributing to fluid and adaptive anticipation of and reaction to future events (Mast et al., 2014; Takakusaki, 2017).

Although the vestibular system’s unusually widespread influences on the brain support the notion that it contributes toward cognitive and emotional processes in health and disease (Balaban et al., 2011; Hoffe and Balaban, 2011; Ferrè and Haggard, 2020), its consideration in trauma-related conditions is relatively novel. Where vestibular signals are in a unique position to directly modulate both low-level multisensory processing as well as higher-order bodily representations, it may be uniquely positioned to contribute to the widespread alterations in sensory modulation and one’s orientation toward reality seen in dissociative episodes and flashbacks. In PTSD, it is well-known that hypervigilance, exaggerated startle response, and pervasive negative emotionality plagues everyday existence. Traumatized individuals feel unsafe or under threat when confronted with a barrage of sensory signals within the context of a currently or previously threatening situation, resulting in a hyperfocus on potentially dangerous exteroceptive stimuli. Although the mechanisms are less than clear, the role of vestibular information in modulating multisensory inputs may be faulty in PTSD and contribute toward state-dependent sensory hyper and hypo-sensitivities to exteroceptive stimuli. Disturbed temporal binding of sensory information engenders perceptual chaos and lack of coherence, which may lead to bodily disconnect (“I feel dead inside”) as well as states of hypervigilance (“I have to be on guard all of the time”) (Foa et al., 1999). Importantly, significant variation in vestibular nuclei connectivity between PTSD and PTSD + DS has been shown (Harricharan et al., 2017), with PTSD + DS exhibiting significantly decreased connectivity with the TPJ and cortical regions. Given the dissociative symptomology within this population, a lack of vestibular innervation of regions involved in bodily consciousness may result in frequent and profound detachment from the body and/or environment manifesting as depersonalization and derealization symptoms, respectively.

### The somatosensory system

The somatosensory system is comprised of the skin, muscles, and joints which detect light touch, deep pressure, pain, temperature, and proprioceptive input. The somatosensory system contributes toward both interoceptive and exteroceptive processing due to its perception of stimuli originating inside and outside of the body, impacting higher order awareness of the physiological state of the body and the immediate external environment, respectively (Abraira and Ginty, 2013). Tactile and proprioceptive information provide crucial information about the body in physical space and as it relates to the environment and others. Given that tactile input has a broad impact on bodily function and percept, ranging from simple reflexes to arousal regulation to complex social processes, it is also highly influential in numerous central nervous system processes (Ayres, 1972; McGlone et al., 2014; Lane et al., 2019).

Touch is detected through specialized cutaneous mechanoreceptors (Gillespie and Walker, 2001; del Valle et al., 2012) and can be further delineated into non-affective and affective touch (McGlone et al., 2014). Newborns respond differentially to affective and non-affective touch (della Longa et al., 2021), indicating that these two channels operate separately from birth. Discriminative or non-affective touch travels via myelinated A-beta afferents projecting to the primary somatosensory cortex (S1) (Olausson et al., 2010; Manzotti et al., 2019). It perceives and localizes the shape and surface structure of objects, guides motor actions, and provides feedback from the body’s active engagement with its environment (McGlone et al., 2007). This pathway is also important for postural schema, which relates to the perceptual representation of the size and shape of the body (Longo et al., 2009; Serino and Haggard, 2010). Deep touch pressure and vibration stimulate Pacinian corpuscles, another type of mechanoreceptor deeper in the skin (Bell et al., 1994), which have effects on autonomic arousal; deep pressure for even brief time periods have resulted in decreased sympathetic arousal (Reynolds et al., 2015) while moderate deep pressure massage has been associated with increased parasympathetic activity (Diego and Field, 2009). Pain and temperature are detected through differentiated high-threshold mechanoreceptors which travel through the unmyelinated spinothalamic tract. The spinothalamic tract travels from the body to the thalamus via the reticular formation, influencing arousal and behavior in response to potential harm or danger (Fregoso et al., 2019).

Affective touch is experienced through the activation of low-threshold tactile mechanoreceptors (C-LTMR) which respond preferentially to gentle, stroking, skin-temperature touch on the hairy regions of the skin (Löken et al., 2009; Morrison et al., 2011; McGlone et al., 2014). Unmyelinated “CT-afferent” pathways (Ackerley et al., 2014; Björnsdotter et al., 2014) transmit affective touch and selectively activate the posterior insula (PI) (Olausson et al., 2002, 2008; Davidovic et al., 2019; Tuulari et al., 2019), a cortical region involved in awareness of the body’s physiological status (Craig, 2002; Olausson et al., 2010). Stimulation of CT-afferent pathways relates to positive affect (Pawling et al., 2017), relief of physical and emotional pain (von Mohr et al., 2017, 2018), reduction of heart rate (Ditzen et al., 2007; Fairhurst et al., 2014; Manzotti et al., 2019), improvement of interoceptive awareness (Crucianelli et al., 2018; Crucianelli and Ehrsson, 2022), attenuation of anxiety/defensive responses (Liu et al., 2018; Walker et al., 2020; Wang et al., 2020), and seeking of social contact as mediated by oxytocin release (Olausson et al., 2002; Morrison et al., 2010; Brzozowska et al., 2022). In rat studies, maternal care is indexed by maternal grooming (licking) behavior toward pups, which has repeatedly been shown to attenuate stress and regulate the development of hormonal, emotional, and cognitive responses to stressors (Caldji et al., 1998; Liu et al., 2000; Meaney, 2001). Increased receptor levels for oxytocin are reported with repeated provision of tactile-rich grooming stimuli in young rats (Francis et al., 2000; Champagne et al., 2001). Importantly, CT-afferent pathway stimulation simulating maternal grooming is sufficient for enhancing oxytocin neuronal firing and promoting prosocial behaviors when rats reached adulthood, while touch deprivation leads to social isolation and diminished preference for social contexts (Bourinet et al., 2021; Yu et al., 2022). Therefore, affective touch is a medium through which we display social concern and attenuate stress, non-verbally communicating our presence in real time and space with another (Gallace and Spence, 2010; Cascio et al., 2019; Ciaunica et al., 2021a; Bohic and Abraira, 2022).

While tactile receptors provide information about the body’s contact with its environment and others, proprioceptors inform where the physical body is in space. Proprioception is detected through Golgi tendon bodies in the joints and muscle spindle fibers in muscle tissue, and informs of joint position and muscle fiber recruitment (Tuthill and Azim, 2018). Skin mechanoreceptors also contribute to proprioception when skin stretches in response to changes in joint angle (Taylor, 2009). The integration of proprioceptive input from the neck, trunk, and limbs with vestibular input at the brainstem level is crucial to inform us whether just our head, our trunk and head, or our whole body has moved in space. Vibration applied to tendons elicits illusory limb motion and dimensionality, impacting body schema and balance (Eklund, 1972; Goodwin et al., 1972; Lackner, 1988; McGlone et al., 2002; Romaiguère et al., 2003; McIntyre and Seizova-Cajic, 2007; Seizova-Cajic and Sachtler, 2007; Fasold et al., 2008; Palluel et al., 2011) and illuminating the importance of proprioception for our sense of stability and constancy with regard to the body in space (Taylor, 2009).

**FIGURE 2:**
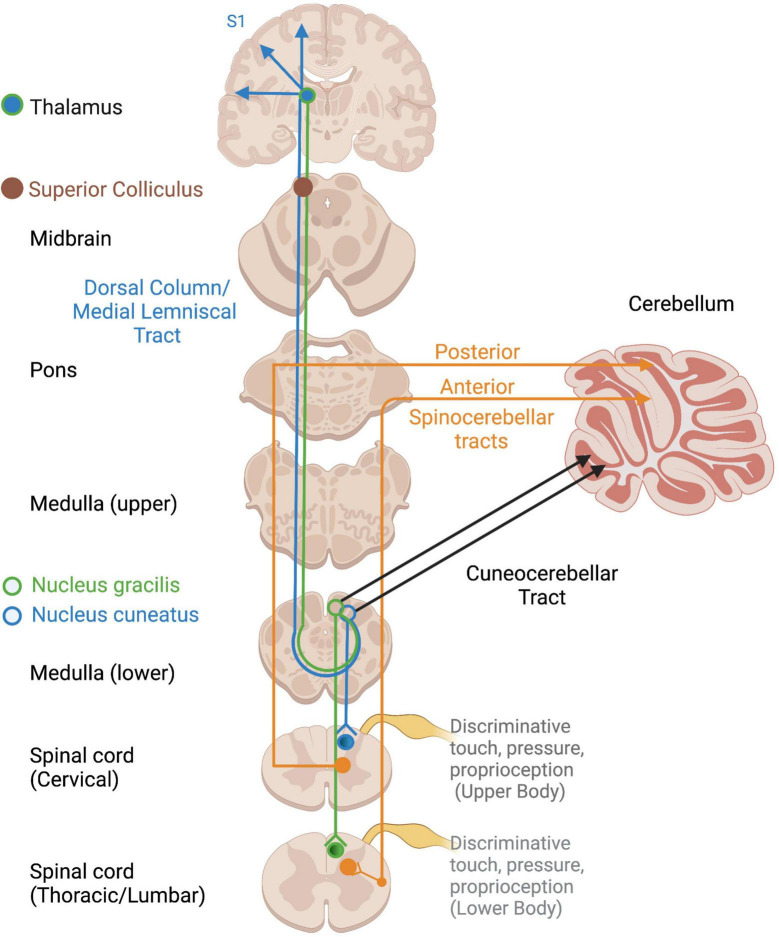
Ascending cortical and cerebellar somatosensory tracts. Open circles reflect terminations of sensory tracts, after which projections take various pathways. DC/ML, Dorsal column/Medial lemniscus; VPL, ventral posterior lateral nucleus of thalamus.

Several somatosensory tracts exist depending on the location and type of input (Figure 2). Non-affective tactile and proprioceptive input project along the dorsal column-medial lemniscal pathway to the superior colliculus, thalamus, and S1, where a representation of the physical body is constructed (Edwards et al., 1979; Kosinski et al., 1988; Taylor, 2009). Proprioceptive input travels to the cerebellum via the spinocerebellar and cuneocerebellar tracts for motor coordination, motoric adaptations to environmental demands, and arousal regulation via connections with the RAS (Ayres, 1972), a structure within the reticular formation which spans the brainstem from the medulla to the mesencephalon (French and Magoun, 1952; Wijdicks, 2019). Proprioception also projects to the cortex via the thalamus to allow for awareness and voluntary control over the body’s position and movements (Taylor, 2009; Tuthill and Azim, 2018). It also innervates the brainstem, including the vestibular nuclei, suggesting widespread influences and elusiveness with regard a primary processing area.

**FIGURE 3:**
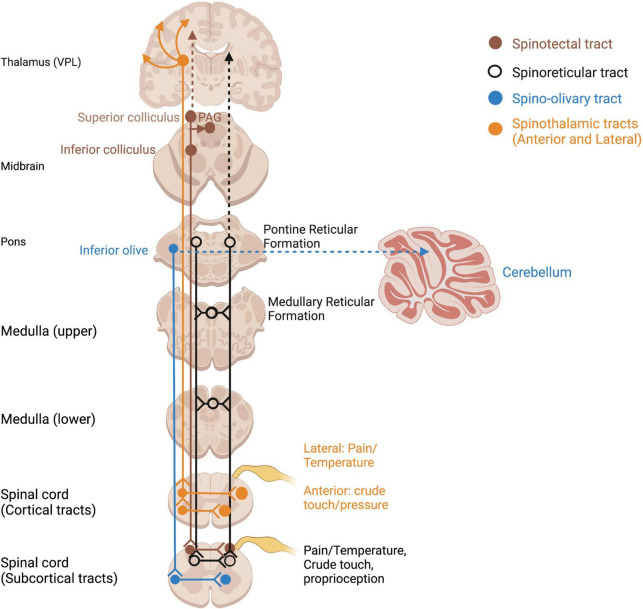
Ascending spinothalamic and subcortical somatosensory tracts. Open circles reflect terminations of sensory tracts, after which projections take various pathways. PAG, periaqueductal gray; VPL, ventral posterior lateral nucleus of thalamus.

Somatosensory information of homeostatic relevance (pain, temperature, affective touch) is conveyed by unmyelinated, phylogenetically older afferent tracts (Marshall and McGlone, 2020). Pain and temperature detected through nociceptors and thermoreceptors travel in an anatomically distinct pathway to the thalamus and S1 via smaller-diameter, unmyelinated axons of the spinothalamic tract (Taylor, 2009) (Figure 3). Given the vast literature in pain processing and our focus on somatosensory contributions to embodiment and self, it is beyond the scope of this paper and will not be covered in this review (for a review of trauma-related pain disorders, see Brennstuhl et al., 2015; Kind and Otis, 2019). Several pathways which innervate subcortical regions such as the inferior and superior colliculi and reticular formation also carry somatosensory and nociceptive information and may be evolutionary relics of phylogenetically primitive animals without neocortices (Figure 3). Direct somatosensory input to the midbrain tectum via the spinotectal tract, as well as the reticular formation via the spinoreticular tract, is an important consideration for how somatosensory input mediates multisensory integration.

**FIGURE 4:**
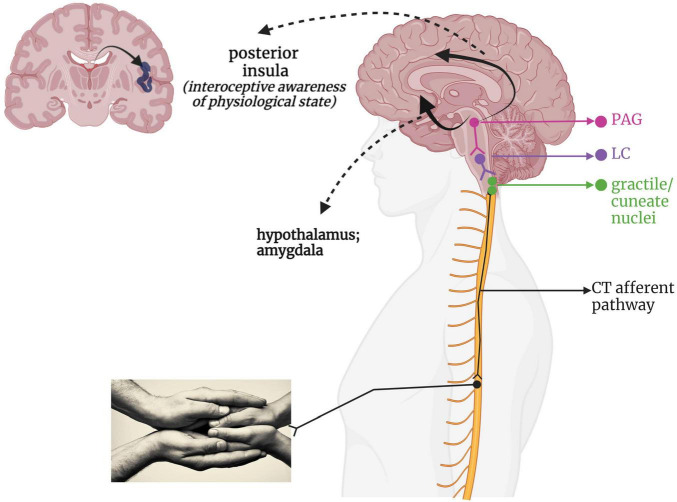
A putative CT-afferent pathway. Light stroking touch to the skin first arrives at the gracile and cuneate nuclei within the brainstem, which interacts with the LC within the RAS to influence arousal. This information flows onward to the midbrain PAG for determination of affective valence and survival relevance. This integrated information then is relayed via a ventral stream to the amygdala and hypothalamus for further emotional processing and endocrine responses, as well as a dorsal stream providing indirect connections to the cingulate cortex and posterior insula. LC, locus coeruleus; PAG, periaqueductal gray; PI, posterior insula; RAS, reticular activating system.

Given its recent discovery as a separate somatosensory phenomenon, the neural pathway taken by affective touch remains speculative. Figure 4 presents a hypothetical CT-afferent pathway based on its innervation of the posterior insula in humans (Olausson et al., 2002, 2008; Björnsdotter et al., 2009; McGlone et al., 2014; Jönsson et al., 2018; Tuulari et al., 2019) and the periaqueductal gray (PAG) and hypothalamus in mice (Yu et al., 2022). Indeed, damage to the right insula reduces pleasantness ratings of CT-optimized touch (Kirsch et al., 2020). Some researchers have categorized affective touch as an interoceptive sensation as it conveys information about the body’s internal state and contributes to experiences of emotion and physiological state (Björnsdotter et al., 2014; Marshall and McGlone, 2020); however, it should be noted that others regard any externally applied stimulus as exteroceptive, reserving the term interoception for internally generated feedback informing upon the state of the body (Craig, 2002; Critchley and Garfinkel, 2017; Allen, 2020). Interestingly, while CT-optimized touch is perceived as highly pleasant in healthy individuals (Löken et al., 2009), individuals with PTSD and/or history of trauma can report it as feeling unpleasant and intense (Badura-Brack et al., 2015; Strauss et al., 2019; Maier et al., 2020). Individuals who experienced a lack of positively valenced, affectionate touch in childhood, such as in cases of neglect and abuse, present with blunted sensitivity to the social value of touch (Devine et al., 2020) and lower blood plasma oxytocin levels (Heim et al., 2009; Donadon et al., 2018), which may be tied to disruptions in CT-afferent driven neurodevelopment.

## Multisensory integration: Phylogenetic and ontogenetic development

*“Most full-grown trees have a remarkable canopy of branches and leaves that interact dynamically with the environment. However, the spreading branches cannot function or survive without the nourishment and support they receive from the roots and trunk. We may appreciate the tree for its spreading leaves, but our understanding must begin with the seed, the roots, and the emerging trunk*.” (Panksepp, 1998, p. 302).

An optimal understanding of how trauma impacts somatic sensory processing requires a deeper appreciation of the evolutionary neurobiology of the mammalian brain. The spinal cord and brainstem are the first central nervous system structures to evolve in vertebrates and develop in the mammalian womb, and are centrally concerned with survival. Likewise, the vestibular and somatosensory systems are known to be the phylogenetically and ontogenetically oldest systems initially concerned with survival-related processes. These somatic systems are more foundational than primarily exteroceptive visual and auditory systems, which develop as the next sensory “layer” (Ayres, 1972; Roley et al., 2007). With mammalian evolution came the development of additional social-emotional circuitry to ensure offspring nurturance and social relationship maintenance for survival purposes. The development of limbic and neocortical structures afforded the ability to act upon the environment as opposed to passively respond to sensory stimuli, and initiate and maintain prosocial contact (Panksepp and Northoff, 2009). Piaget (1952) and Herrick (1956) postulated that the brain retained its older structures and reorganized them with each cortically directed evolutionary step, similar to the ontogenetic pattern of neurodevelopment. Maclean’s (1985) triune brain theory is in agreeance, suggesting the brain to be a phylogenetically layered system that began with the “reptilian” or subcortical brain. Upon the foundation of subcortical structures developed the limbic system, now nestled within the cortical midline and heavily involved in emotion and self-related processing (Panksepp and Northoff, 2009). The final neocortical layer engenders higher cognitive capacities and behavioral flexibility (Maclean, 1985). Despite general focus on the neocortex as our evolved and “smart” master control center, sub-cortical regions maintain a critical role in primitive survival functions and sensorimotor control. This pre-reflective, multisensory integrative region may be most impacted by threats to survival, giving rise to deficits in regulatory capacities and post-traumatic symptoms (Perry and Hambrick, 2008; Perry, 2019). A neurodevelopmental and phylogenetic framework contextualizes the disparity between which aspects of trauma can be articulated and which remain trapped or pre-reflectively experienced by the brainstem and body.

### Phylogenetic development

#### Vestibular system

Even the most primitive of mobile organisms must recoil from noxious stimuli, maintain motoric control and orientation while moving about in the environment, and respond with either motion or defensive posturing to external threats. Vestibular function was established in ancient vertebrates and has remained largely unchanged from fish to humans, as opposed to auditory structures and networks which adapted to terrestrial life and mammalian dynamics (Fritzsch et al., 2014; Lipovsek and Wingate, 2018). The ipsilateral vestibulospinal and reticulospinal tracts are the most ancient motor tracts in the human body, dating back to a time before forebrain lateralization (Vulliemoz et al., 2005; Mora et al., 2019). The vestibulospinal pathway originates at the vestibular nuclei, controlling posture and balance while regulating muscle tone (Akaike, 1983; Sengul and Watson, 2015). For instance, the linear acceleration sensed while stumbling sends excitatory input down the vestibulospinal tract to engage the extensor musculature responsible for equilibrium responses and postural control, reflexively protecting the head and body from impact (Shinoda et al., 2006; McCall et al., 2017). The reticulospinal tract originates within the pontine and medullary reticular formation and supports anticipatory postural responses, regulation of muscle tone, and autonomic regulation (Sengul and Watson, 2015). Efficient reflexive responses of the body have evolved to maintain efficient movement through space, giving eventual rise to a sense of agency over purposeful bodily action.

#### Somatosensory system

The function of skin tissue is in a sense analogous to that of brain tissue in that both are designed to be an interface between the body and the outside world (Panksepp, 1998). Phylogenetically, the receipt of touch from another is a ubiquitous experience for both aquatic and terrestrial species. Fish that seek cleaning of ectoparasites and tactile stimulation from “cleaner fish” have lower cortisol levels as well as preserved motivation to visit a cleaner fish “replica” for tactile stimulation in and of itself, showing that tactile stimulation alone imparts physiological changes (Soares et al., 2011). A phylogenetically older, aquatic touch system may have been co-opted by social mammals in keeping with evolutionary continuity to preserve a tactile-mediated sense of safety and security (Schirmer et al., 2013). This corresponds with the unmyelinated, small-diameter axon morphology of CT-afferent tracts in humans, suggesting phylogenetic precociousness. Ubiquitously, tactile stimuli functions to relieve stress and enhance health in mammals and non-mammals alike (Caldji et al., 1998; Liu et al., 2000; Meaney, 2001; Raussi et al., 2003; Schmied et al., 2010; Soares et al., 2011) to the extent that evolution has provided specialized receptors and pathways. This suggests that tactile facilitation of physiological regulation and recovery from fear has roots in our evolutionary beginnings, and thus has implications for how primal fear and survival-based circuitry may be altered by trauma.

### Ontogenetic development

#### Vestibular system

The vestibular system is the first sensory system to begin development *in utero* (Sidebotham, 1988; Blayney, 1997), and the morphology of the vestibular apparatus is mature within the first month of gestation (Nandi and Luxon, 2008; Jamon, 2014). The vestibulocochlear nerve is the first cranial nerve to complete myelination and the system is fully developed by the eighth month of intrauterine life (Blayney, 1997; Nandi and Luxon, 2008). Although morphologically mature, developmental changes in vestibular connectivity with the cerebellum and cortex continue through early life as children move to learn and grow (Lane et al., 2019). Initially, the fetus detects the mother’s movement in the womb, with movement becoming less externally controlled and more self-initiated with development. The neurodevelopmentally healthy child has a strong instinctual drive to master bodily control against gravity (von Hofsten, 2007), from the repetitive battles for head control in infancy through the joyful tests of balance on the playground in early childhood. This increase in bodily control against gravity develops in tandem with the child’s sense of agency, or sense that they are in control of their body as an object capable of intentional action. We suggest that the detection of gravity provides an anchor for affective-somatic instinctual happenings, an idea informed by the ontogenetic primacy given to the vestibular system’s development in conjunction with its widespread connectivity within the central nervous system.

#### Somatosensory system

The source of both the skin and the nervous system is the embryonic ectoderm, which differentiates into the surface ectoderm and neuroectoderm, respectively (Montonen et al., 1998; Bond et al., 2012). Fetal somatosensory receptor density is exuberant compared to that of an adult (Ashwell and Waite, 2012), suggesting an essential role in driving neurodevelopment. The fetus responds to touch as early as 8 weeks (Lecanuet and Schaal, 2002; Hadders-Algra, 2007) and elicits self-initiated movements by 12 weeks (Kurjak et al., 2008). The fetal somatosensory system is directly and frequently stimulated by movement of the amniotic fluid over fetal lanugo hairs (Bystrova, 2009; Sasaki et al., 2013), as well as through self-touch (Piontelli, 2015) and self-motion within the confined space of the womb (Marx and Nagy, 2015). These sensory-motor interactions occur well before the infant is born to interact with the outside world. The experience of pleasantness from affective touch has been linked with improved autonomic regulation in newborn humans and animals (Morrison, 2016; van Puyvelde et al., 2019; Farroni et al., 2022), as young children need to be touched and swaddled to co-regulate their emotional worlds. While breastfeeding duration has been associated with improved cognitive development in children even when controlling for socioeconomic position (Pereyra-Elías et al., 2022), the degree that somatosensory input coupled with oxytocin release plays a role is indeterminate yet intriguing. Lastly, affective tactile interactions create a perceptual and physical boundary between self and other within the first months of extrauterine life and beyond (Farroni et al., 2022).

### Somatic sensory contributions to secure and insecure attachment

**FIGURE 5:**
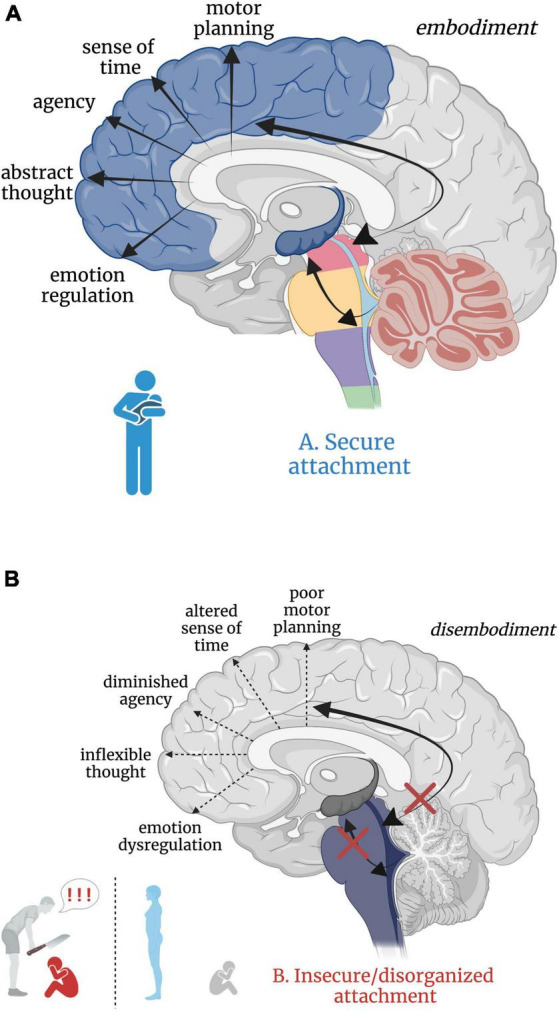
**(A,B)** Good versus poor somatic sensory integration during development with a secure versus insecure attachment figure, respectively. **(A)** Healthy brainstem level multisensory integration as a result of attuned, nurturing, somatic sensory-rich caregiving provides the foundation for higher-order limbic and cortical capacities. **(B)** An overwhelmed or malnourished brainstem due to abusive or neglectful caregiving. The child does not receive comforting touch or attuned rhythmical movement with a safe affective caregiver. Somatic sensory experiences are paired with unsafe or insecure attachment relationships, which has cascading aversive effects on higher-order limbic and cortical capacities.

Attachment theory (Bowlby, 1973) posits that humans have an inborn neurobiological system in place that seeks proximity and care from an attachment figure in times of physical and emotional need, resulting in an ingrained sense of security, safety, and acceptance (Schore, 2003; Mikulincer et al., 2015). Inherently, the development of secure attachment bonds with a primary caregiver requires soothing and comforting sensory input. Somatic “co-embodiment” with the mother forms the basis for an emerging sense of self-embodiment in the young child (Ciaunica et al., 2021a). During uterine development, the fetus experiences co-embodiment on a sensory level in that the fetus’ physical and regulatory developmental needs are dependent upon the mother and intrauterine sensory experiences during prenatal development (Ponzo et al., 2018; Ciaunica et al., 2021a). In a secure attachment relationship, an infant continues to be co-regulated post-natally by an attuned caregiver through somatic sensory experiences such as rocking, swaddling and bodily contact. The caregiver intuitively problem solves which rhythmical patterns of movement and touch will calm their child’s distress and instinctually regulates their child’s physiological state and emotional world until they can self-soothe. Here, somatic sensory input is embedded in the manipulation and regulation of arousal (Balaban and Porter, 1998; Yates and Miller, 1998; Manzotti et al., 2019) given its connections with and integration at the RAS. Indeed, increased frequency of linear and rhythmical rocking attenuates arousal in infants (Vrugt and Pederson, 1973). This caregiver-scaffolded regulation of the infant, termed co-regulation or co-homeostasis (Ciaunica et al., 2021a), not only assists the infant in the moment, but also creates a secure, reliable, and safe attachment bond. It is through this physical and physiological attunement that children sense that they are protected, cherished, and safe. Speculatively, this foundation solidifies the child’s connection to the earth and confidence to emerge from the co-regulated bond, eager to explore as a separate and capable organism worthy of the social connection crucial for survival and reproduction later in life. In secure attachment, the higher reaches of the child’s limbic system and neocortex develop upon the foundation of an integrated and modulated subcortical layer (Figure 5A).

Insecure/disorganized attachment results from early experiences with negatively valenced, absent, or inconsistent physical and/or emotional interpersonal interactions. Here, physical and emotional harm occurs through active abuse, withdrawal of physical comfort, or somatic sensory experience interacting with negative or withdrawn affect. Though there are distinct differences between anxious-insecure, avoidant-insecure, and disorganized attachment patterns, it is beyond the scope of this paper (instead see Bowlby, 1973; Ainsworth et al., 1978; Paetzold et al., 2015; Beeney et al., 2017) and thus we consider all non-secure attachment patterns here as ‘insecure’. Insecure attachment patterns with primary caregivers are posited to manifest as dissociative response patterns (i.e. freezing/tonic immobility) in adulthood (Ogawa et al., 1997; Schore, 2003; Lyons-Ruth et al., 2006), where emotional abuse or neglect is most predictive of dissociation (Sar et al., 2009). Those who experience early life abuse or neglect from a caregiver may develop dissociative response patterns as an adaptive means of escape from childhood stress and show reduced oxytocin levels in adulthood (Heim et al., 2009; Opacka-Juffry and Mohiyeddini, 2012; Bertsch et al., 2013), diminishing protection from stress and reducing propensity to form close relationships. However, the relationship between severity of early life trauma, genetics, and oxytocin has proved complicated, warranting further investigation (Chatzittofis et al., 2014; Myers et al., 2014; for a review, see Donadon et al., 2018). From a neurobiological perspective, subcortical sensory integrative regions crucial for higher limbic and cortical development are overwhelmed by alarming and distressing sensory input, starved for sensory-affective nourishment, or conflicted by the inconsistent provision and withdrawal of sensory-affective nourishment in abusive, neglectful, or disorganized caregiver relationships, respectively. We hypothesize that insecure attachment has both a somatic sensory component and a cascading effect on ontogenetic development of the higher reaches of the brain important for emotion regulation, motor planning, abstract thought, sense of time, agency, curiosity, and dynamic social relationships (Figure 5B). Though speculative, aberrant somatic sensory integration correlated with insecure attachment patterning may set the stage for later psychiatric dysfunction, providing insight into the high correspondence between early life traumatization with insecure attachment and the later development of psychopathology and/or PTSD (Roche et al., 1999; Besser and Neria, 2012; Mikulincer et al., 2015; Ogle et al., 2015; Woodhouse et al., 2015; for a review, see Marshall and Frazier, 2019).

## Neurobiology of somatic sensory processing: Role of the brainstem and midbrain in arousal, defensive responding, and trauma-related symptomology

While subcortical brain regions integrate somatic and exteroceptive sensory information to create a coherent body representation, they are implicated in evolutionarily conserved threat detection and response circuitry (Darwin, 1872; Adamo, 2012; Tovote et al., 2016; for a review, see Terpou et al., 2019b) as well as in instinctual social interactions for mammalian survival. As the somatic sensory systems give rise to a general experience of the physical body while preserving its safety and viability, they are highly implicated in the body’s response to traumatic events. Alterations to muscle tonicity protect the body from harm, ranging from immobile rigidity to flaccidity to prevent detection from a predator or the experience of overwhelmingly noxious sensation, respectively. Somatosensory feedback from the peripheral somatosensory system contributes toward safety or threat detection, the latter resulting in the maintenance of a midbrain-mediated, survival-focused loop. We suggest that neurobiological pathways arising from the *soma*, as mediated by vestibular and somatosensory feedback, are principal in integrating incoming exteroceptive sensory stimulation (related to the external environment) and interoceptive stimulation (related to the viscera and internal physiological state) with contextual information regarding safety or potential danger at the level of the midbrain. The direct connections these somatic sensory systems have with brainstem and mesencephalic regions influence a subconscious maintenance of homeostasis and a felt sense of relational security with our surroundings and with others.

### Arousal

Where traumatization is strongly characterized by incapacitated arousal modulation (Pitman et al., 1987; van der Kolk, 1987; Bryant et al., 2000; McMillen et al., 2000; Bryant, 2003; Risser et al., 2006; for a review, see Pole, 2007; Pitman et al., 2012), the sensory-motor contributions toward arousal are crucial to consider. Arousal level is directly influenced by sensory stimuli, and responses to sensory stimuli are influenced by arousal, suggesting an inextricable and bidirectional relationship. The RAS, comprised of numerous nuclei, generates raw arousal and “alarm” mainly within the locus coeruleus (LC) and pedunculopontine nuclei (PPN) (Berridge and Waterhouse, 2003; Aston-Jones and Cohen, 2005; Horn and Adamzyk, 2012; Pfaff et al., 2012) and has reciprocal interactions with the brainstem vestibular nuclei (Yules et al., 1966; Pompeiano et al., 1984; Manzoni et al., 1989; de Cicco et al., 2018) and somatosensory dorsal column (gracile and cuneate) nuclei (Guzmán-Flores et al., 1962; Odutola, 1977). The appropriate level of attention and alertness required for higher order emotional and cognitive processes is then mediated by the thalamus (LaBerge et al., 1992; Coull, 1997; Portas et al., 1998).

Both hyper- and hypoarousal are common phenomena in traumatized individuals. Hyperarousal results in heightened sensory and emotional reactivity (Pfaff et al., 2012), and falls within the DSM-V diagnostic criteria for PTSD (American Psychiatric Association [APA], 2013). Alternatively, decreased arousal may contribute toward the dampened emotionality and hypo-responsivity to stimuli evident in the dissociative subtype (Frewen and Lanius, 2006; Ludäscher et al., 2007; Sack et al., 2012; D’Andrea et al., 2013; Thome et al., 2019). Individuals with PTSD + DS exhibit altered resting-state functional connectivity (rsFC) between the PPN and the amygdala and ventromedial prefrontal cortex (vmPFC), and rsFC between the PPN and anterior thalamus correlates negatively with derealization/depersonalization symptoms (Thome et al., 2019). While heightened RAS-amygdala and RAS-vmPFC rsFC suggests enhanced threat processing (Liddell et al., 2005; Williams et al., 2006; Lanius et al., 2017), a negative relationship between RAS-thalamus rsFC and dissociative symptomology may indicate disrupted transmission of multimodal sensory information to the cortex. When the latter result is coalesced with findings of diminished vestibular nuclei rsFC with cortical regions in PTSD + DS (Harricharan et al., 2017), we converge on a hypothesis that dissociative symptomology is related to cortical-somatic sensory deafferentation, resulting in hypo-arousal and altered perceptual awareness of the body and its surroundings. Stress or trauma recall-induced dissociation has been linked to blunted psychophysiological arousal responses in traumatized individuals with high dissociative symptoms (Griffin et al., 1997; Koopman et al., 2004; Sack et al., 2012; D’Andrea et al., 2013; for a review, see Boulet et al., 2022), as well as in individuals with psychiatric conditions highly associated with severe early life traumatization including dissociative disorder (DD) (Schäflein et al., 2018), borderline personality disorder (BPD) (Ebner-Priemer et al., 2005; Bichescu-Burian et al., 2018) and dissociative identity disorder (DID) (Williams et al., 2003; see Putnam et al., 1986 for a review). Tendencies toward hyper-arousal and blunted arousal states in PTSD and PTSD + DS, respectively, point toward a psychophysiological manifestation of PTSD symptomology and different patterns of arousal-related neural circuitry in non-dissociative and dissociative trauma-related disorders. Importantly, a blend of intermittent hyper- and hypo-arousal may exist in traumatized individuals, indicative of broadly disrupted arousal modulation patterns that may be difficult to capture in statistical research methodology which centers or averages physiological or self-reported data on arousal.

Arousal can be attenuated through rhythmical linear vestibular stimulation (Vrugt and Pederson, 1973; Pederson, 1975; Lubeck et al., 2020; for a review, see Sandler and Coren, 1981), deep touch pressure (Diego and Field, 2009; Reynolds et al., 2015), and light touch (López-Solà et al., 2019; Reddan et al., 2020) within the context of safety. Individuals seek linear somatic sensory input in the form of walking, cycling, and running to calm their “nerves” or racing thoughts, and touch from a loved one to alleviate distress or improve mood. Conversely, sudden acceleration, angular motion, itching, light crawling touch from a bug, and pain will increase arousal to direct our attention to a potentially precarious or threatening bodily situation. Foundationally, somatic sensory input is primarily concerned with our survival and safety given the potential repercussions of falling on our skull, enduring a poisonous spider bite, or disconnecting from a primary caregiver or protective adult relationship, lending to strong connections with arousal centers. In a suboptimal arousal state, we cannot function or sleep properly, undermining higher-order capacities such as learning, memory, and social cognition (Siegel, 2001).

### Muscle tone, posture, and orientation to threat/connection

**FIGURE 6:**
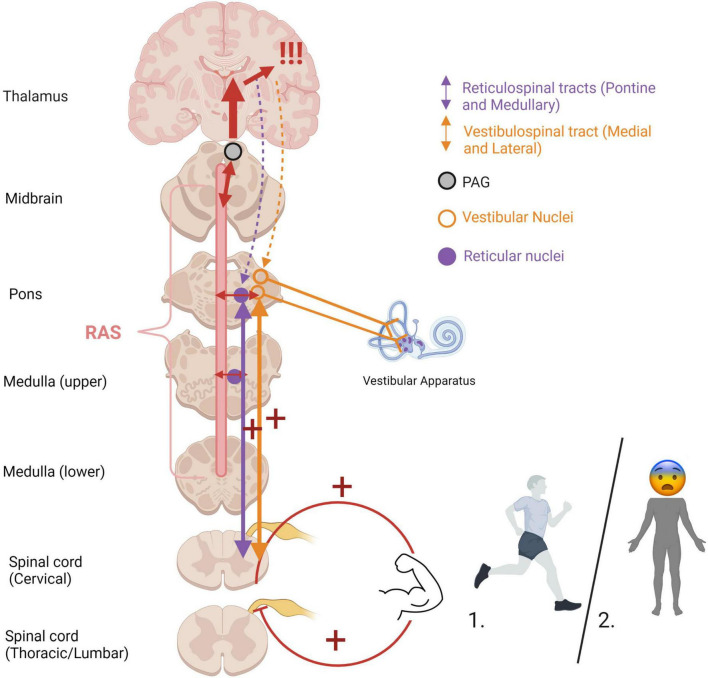
Heightened muscle tonicity during fight/flight (1) and freeze/tonic immobility (2) responses. Overwhelm of subcortical arousal-affective circuitry (RAS and PAG) during traumatic stress cues heightened muscle tonicity, mediated by the vestibular and somatosensory systems. If the body carries out an active survival response, the system receives vestibular and somatosensory feedback from muscle engagement and bodily action. In situations of imminent danger, tonic immobility renders the body rigid, an evolutionarily primitive response to avoid predatory detection. Cortical structures are overaroused, and top-down modulatory influences are weakened (indicated by dashed lines), allowing for primitive brainstem and midbrain regions to dictate defensive responding and heightened muscle tonicity. If the body is prevented from carrying out defensive motor actions (i.e., fighting off, pushing away, running away), it may become pre-disposed to or “stuck” in this defensive posture post-traumatically. RAS, reticular activating system; PAG, periaqueductal gray.

**FIGURE 7:**
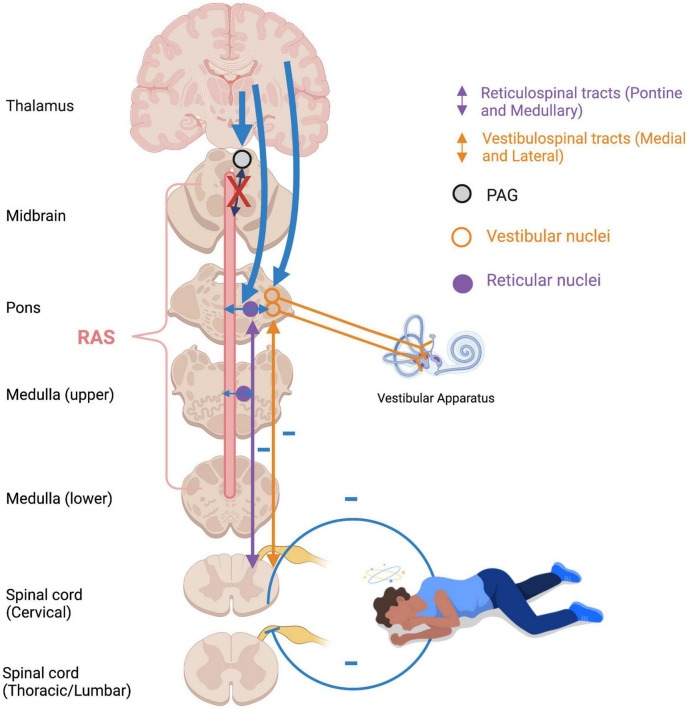
Flaccidity of muscle tone during emotional shutdown/feign death/dissociative responses. Over-modulation of subcortical activation by higher structures (thick blue arrows) as an adaptive defensive response to repeated traumatic stress results in diminished arousal and weakened vestibular efferents to extensor musculature. Feedback from deafferented, flaccid muscles creates a somatosensory-vestibular feedback loop which subconsciously maintains a collapsed, dissociated posture and hypo-aroused state. RAS: reticular activating system; PAG: periaqueductal gray.

An optimal baseline level of muscle tonicity, innervated by the vestibulospinal and reticulospinal tracts, allows for adaptive motor actions and responses. Increased muscle tension occurs during states of high stress or fear to prepare the physical body for fighting or fleeing (Figure 6). If an animal carries out a survival-oriented action to completion, the system receives vestibular and somatosensory feedback. A thwarted or incomplete motor response to a threatening situation may explain how traumatized individuals often experience their body reflexively enters exaggerated reactive motor patterns and/or defensive states such as tonic immobility or collapse in the face of everyday stressors or trauma triggers. Exaggeratedly heightened tonicity and decreased postural sway create a tonic immobility or “freeze” response, exhibited by animals (Webster et al., 1981; Fleischmann and Urca, 1988; Porro and Carli, 1988) and humans (Suarez and Gallup, 1979; Heidt et al., 2005; Marx et al., 2008) who experience imminent threat (for a review, see Terpou et al., 2019b). This response is mediated by somatosensory and vestibular feedback (Klemm, 1971; Gallup, 1977; Fleischmann and Urca, 1988), and is hypothesized to deter predators who are wired to detect motion in their prey and enable the animal to monitor its environment and flee if necessary (Kozlowska et al., 2015). Tonic immobility has been reported by study participants with PTSD under traumatic memory recall (Volchan et al., 2011; Fragkaki et al., 2016; de Kleine et al., 2018), suggesting that traumatic memories remain connected to subconscious postural responses. Tonic immobility can be elicited in animals using species-specific forms of repetitive tactile or vestibular stimulation, including inversion and somatosensory restraint (Klemm, 1971; Webster et al., 1981; Fleischmann and Urca, 1988; Porro and Carli, 1988; for a review, see Kozlowska et al., 2015). Likewise, tonic immobility in humans is more common under fear-inducing conditions of somatosensory restraint, such as rape (Suarez and Gallup, 1979; Heidt et al., 2005; Marx et al., 2008). Alternatively, hypo-arousal and decreased muscle tone occur during emotional shut-down or feign death as a passive defensive response to inescapable or prolonged threat (Hofer, 1970; Depaulis et al., 1994; Bracha, 2004). Here, drastically reduced arousal and tonicity diminish the conscious experience of further injury and psychic distress during inevitable or prolonged attack, sometimes to the point of an out-of-body experience or complete loss of consciousness (Figure 7). These passive responses are likely when traumatization is chronic, or during childhood where the victim is ill-equipped for self-defense and the perceived potential of escape is low (Nijenhuis et al., 1998b; van der Kolk, 2003; Schauer and Elbert, 2010; Krammer et al., 2016; Kratzer et al., 2018; for a review, see Foa and Hearst-Ikeda, 1996). Accordingly, those with trauma-related dissociative disorders are thought to exhibit top-down overmodulation of subcortical brain activity (Lanius et al., 2010b; Nicholson et al., 2017; Terpou et al., 2020), which putatively suppresses somatic sensory information from reaching higher-order regions involved in its integration into awareness.

The superior colliculi (SC) of the midbrain tectum (mesencephalon) are powerful multisensory integrative centers containing layered topographical maps of the visual, auditory, and somatosensory systems (Meredith et al., 1992; Cuppini et al., 2018; Basso et al., 2021). The SC are best known for their involvement in subconscious orienting responses to salient visual stimuli (Dean et al., 1989; Grantyn et al., 2005; May, 2006; Horn and Adamzyk, 2012). They send afferents to oculomotor neurons, the pontine and medullary reticular formation, and cervical and upper thoracic motor neurons to orient the eyes, head and neck toward or away from stimuli (May and Porter, 1992; Scudder et al., 1996; May, 2006). Importantly, the SC receive ascending somatosensory feedback from the spinotectal (spinomesencephalic) tract (refer back to Figure 3) and have reciprocal connections with the vestibular nuclei innervating head and neck musculature (Rubelowski et al., 2013; Leong et al., 2019). Vestibular and proprioceptive inputs to the SC are required to appropriately gauge the force and speed required for a rapid yet ergonomically sound orienting response. Pertinently, this orienting response is geared toward instinctually relevant sensory stimuli, including threatening *and* nurturing interpersonal contact (Dean et al., 1989), playing a major role in approach and defense/withdrawal responses in early attachment and traumatic interpersonal dynamics (Corrigan and Christie-Sands, 2020). Aberrant rsFC of the SC has been shown in PTSD and PTSD + DS, suggesting different patterns of defensive responding in dissociative individuals (Olivé et al., 2018).

Taken together, heavy involvement of somatic sensory processing is likely in defensive posturing and motoric responses to traumatic events. Post-traumatically, the somatic sensory systems may prioritize their role of self-protection and defense, superseding their roles in orienting toward social contacts and maintaining a coherent multisensory experience. Further, primal defensive responses may remain online as a person feels “stuck” between an activated (or hypo-activated) autonomic response and the completion of self-protective action (Ogden et al., 2006). When the body is unable to self-protect (or protect another), the individual’s sense of agency is attacked and self-trust falters. When the execution of movement to self- or other-protect is disrupted, states of psychic anger or rumination may be disconnected from the body, or bodily activation may be disconnected from awareness (Corrigan and Christie-Sands, 2020). This disrupted subcortico-cortical information flow engenders pervasive senses of defectiveness and defenselessness, and may contribute to unintegrated traumatic memory manifesting at somatic or affective levels (Janet, 1889; Nijenhuis et al., 1998b).

### The periaqueductal gray, affect, and defensive responses

The periaqueductal gray (PAG), a region of gray matter surrounding the cerebral aqueduct of the midbrain, acts as both a sensory relay and a sensory-affective integrative center that coordinates subcortical defense or approach behaviors in light of contextual information (Bandler and Depaulis, 1991; De Oca et al., 1998; Panksepp, 1998; Carrive and Morgan, 2012; for a review, see Kozlowska et al., 2015). The dorsal PAG plays a key role in fight/flight defensive responses (Brandão and Lovick, 2019), while the lateral/ventrolateral PAG is indicated in passive defensive responding including tonic immobility and emotional shutdown/collapse (Keay and Bandler, 2001; Lovick and Bandler, 2005; Koutsikou et al., 2014) as well as affective touch-mediated social behaviors in mice (Yu et al., 2022). The PAG receives sensory information from the pontine and medullary reticular formation, superior colliculus, and inferior colliculus among other brainstem regions, and integrates this with positive or negative affective valence from descending limbic inputs from the amygdala, hypothalamus, insula, and posterior cingulate (Carrive and Morgan, 2012). Affect laden sensory information then projects to cortical structures via the thalamus and hypothalamus (Damasio, 1998; Buck, 1999; Northoff et al., 2006; Hurley et al., 2010; Carrive and Morgan, 2012; Koelsch et al., 2015). Importantly, the PAG relays information to the insula and anterior cingulate cortex, giving rise to the salience network (SN) (see section “PAG and the salience network”) and contributing toward interoceptive and emotional awareness (Craig, 2003, 2009; Critchley et al., 2004). Top-down projections to the PAG serve to modulate arousal after contextual appraisal and maintain homeostasis (Critchley et al., 2004; Wiens, 2005; Barrett and Simmons, 2015). In particular, the amygdala participates in conditioned fear responses via a descending amygdalo-hypothalamo-PAG circuit, which Panksepp (2000) defined as the FEAR system, one of the basic raw affective systems engendered at the PAG. Top-down influences from the hypothalamus may also be involved in the conditioning of positive emotional experiences such as nurturance (Panksepp and Trevarthen, 2009; Yu et al., 2022). Overall, the PAG elaborates incoming sensory stimuli with affect to ascertain whether an environment or another animal is safe or threatening. For instance, light touch from a romantic partner at home will elicit a very different physiological and emotional response than light touch while walking alone in a dark alley. Likewise, a previous experience of assault in a dark alley will render the context of an alleyway or a grab to the arm sufficient to activate amygdalo-hypothalamo-PAG FEAR circuitry. Taken together, sensitization may occur where a stimulus or constellation of stimuli experienced during a previous event re-invokes a somatic and emotional response, facilitating either defensive or palliative, pro-social behavior (Anagnostaras and Robinson, 1996; Yehuda et al., 1998; Milligan-Saville et al., 2017; for a review, see Perry et al., 1995).

### Periaqueductal gray and the salience network

The PAG is a node of the salience network (SN), an intrinsic connectivity network that activates when presented with unexpected, intense, or instinctually relevant sensory or emotional input (Menon, 2015). The SN is activated when lower-level sensory input meets a threshold for orienting our attention. The SN also plays a role in adaptive switching between the central executive network (CEN), involved in focused attentional states, and the default mode network (DMN), active during introspective self-referential thought and social emotional processes (Amodio and Frith, 2006; Sridharan et al., 2008; Menon and Uddin, 2010; Spreng and Grady, 2010; Menon, 2015). Individuals with PTSD have shown hyperactivation and hyperconnectivity of the SN (Thome et al., 2014; Abdallah et al., 2019; for a review, see Akiki et al., 2017), as well as diminished coherence across the DMN (Bluhm et al., 2009; Lanius et al., 2010a; Sripada et al., 2012; for a review, see Akiki et al., 2017; Lanius et al., 2020). Hypothetically, any form of salient sensory stimulation including slight movements, loud noise, or unexpected touch may trigger a heightened arousal response within a quick PAG-mediated defense mechanism short-circuited from receiving higher-order contextualization. Alternatively, those with dissociative symptomology may experience top-down over-modulation of the PAG as a defense mechanism, resulting in hypo-responsivity to salient sensory input and emotional numbness. Individuals with PTSD have shown hyperactivation of the PAG in response to direct eye-contact (Steuwe et al., 2014) as well as to subliminal threat stimuli (Terpou et al., 2019a). Individuals with PTSD show increased rsFC of the PAG with the anterior insula and anterior cingulate, major nodes of the SN (Harricharan et al., 2016). Alternatively, individuals with PTSD + DS exhibited increased v/l PAG functional connectivity with OP2 and TPJ, primary vestibular-multisensory processing regions (Ibitoye et al., 2022) associated with depersonalization symptoms (Lanius et al., 2005; Harricharan et al., 2017).

## Trauma: An assault on the senses

Where a traumatic event poses a perceptual threat to one’s own bodily safety, or that of another, while eliciting feelings of extreme fear, helplessness, or horror (American Psychiatric Association [APA], 2013), it can also be conceptualized as a negatively valenced multisensory experience- an assault on the senses. Any form of traumatic event is a strongly arousing and affective multisensory experience, thus impacting the lower reaches of the brain. For example, a combat soldier experiences searing heat on the skin, a grotesque scene displayed on the retina, and gut-wrenching interoceptive sensations when a comrade is unable to be saved; a rape victim experiences aversive touch, imposed bodily movement and somatosensory restraint; a child beaten by a caregiver feels bodily pain, sees an expression of rage on their caregiver’s face, and senses a racing heart prevented from fleeing. In the case of chronic emotional neglect or abandonment in childhood, the threat on survival exists in the void of predictable, developmentally crucial sensory experiences such as synchronous movement, touch, and warmth. Taken together, brainstem-level multisensory integrative processes, typically grounding our experience in the present environment and physiological state of the body, interact with raw affect at the PAG to create an overwhelming, aversive experience. Whether a highly stressful event can be adaptively managed or modulated may depend upon the individual’s history of multisensory-affectively bound attachment experiences. Given that extreme stress activates an individual’s innate attachment system (Bowlby, 1973), an inability to seek co-regulation from another or engage neural circuitry related to self-regulatory mechanisms due to a history of insecure attachment and somatic sensory disintegration results in persisting trauma-related symptoms. In more extreme cases where the individual experiences tonic immobility or emotional shutdown, a sensory feedback-motor action plan for survival is thwarted and intention to obtain survival needs is decoupled from expected or intended action. This sensory-motor mismatch is experienced by the body as an inadequate response to existential threat (Fischer and Riedesser, 1999; Sar and Öztürk, 2008), and corresponds with a disrupted sense of agency and connectedness to/trust in the body.

### Secure/insecure attachment as a buffer from/predisposition toward trauma-related conditions

**FIGURE 8:**
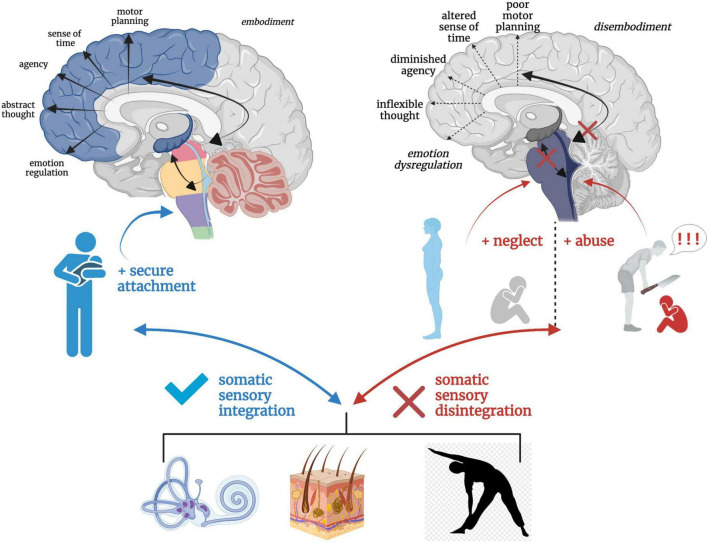
A hierarchical schematic of how somatic sensory systems, mediated by early attachment patterning, provide a foundation for vertical and horizontal integration in the brain. Vestibular, tactile and proprioceptive input drive healthy neurodevelopmental processes and the development of emotion regulation, motor planning, sense of time, agency, and a sense of embodiment when scaffolded by an attuned, secure attachment figure. However, a paucity or lack of safe and nourishing somatic sensory experiences interacting with contexts of abuse and/or neglect results in overwhelmed, fragmented and disintegrated brainstem-level sensory integration, which has cascading effects on higher order capacities of limbic and cortical brain regions.

Solidity in subcortical somatic sensory integration from a secure attachment history may act as a buffer against long-term ramifications of trauma. An individual with a history of secure attachment during critical developmental periods is better equipped to tolerate sensory and affective stress through fluid bottom-up and top-down integrative information processing and return to a solid baseline functioning post-stressor (Dieperink et al., 2001; Mikulincer et al., 2015). However, destabilized somatic sensory processing coupled with an insecure attachment history may render the individual vulnerable to stress and aberrant neural re-wiring in the wake of trauma. Further, traumatic experiences may exacerbate attachment-related insecurities and tendencies toward defensive (as opposed to connection-seeking) responding (Alexander, 1993; Muller and Rosenkranz, 2009; Mikulincer et al., 2015). Individuals with insecure attachment styles exhibit increased severity of psychiatric and PTSD symptoms (Mikulincer et al., 1993, 1999; Solomon et al., 1998; Dieperink et al., 2001; Zakin et al., 2003; Declercq and Willemsen, 2006; Fraley et al., 2006; Ghafoori et al., 2008; Bogaerts et al., 2009; Midolo et al., 2020), and attachment style has shown to be a mediating (Roche et al., 1999; Shapiro and Levendosky, 1999; Twaite and Rodriguez-Srednicki, 2004; Sandberg et al., 2010) and moderating (Sandberg, 2010; Scott and Babcock, 2010) variable between childhood exposure to trauma and psychiatric distress. The repeated inability to fight, flee, or receive physical and emotional safety during critical neurodevelopmental periods leaves hyperactivated brainstem and midbrain regions with no regulatory outlet, leading to cascading effects on higher-order capacities and a vulnerability toward the development of trauma-related dysfunction (Figure 8).

### On defense: Neural circuitry in trauma

When presented with sensory stimulation construed as threatening, less integration or “talking” between neocortical and subcortical regions may result in cortical and subcortical “loops” that take on a life of their own. A subcortical loop from the body to the midbrain and back again short-circuits the frontal regions for efficiency, priming the traumatized individual for rapid defensive responding (Lanius et al., 2017). Cortico-thalamo-cortical circuits may result in self-perpetuating cycles of rumination, obsessive thoughts, and a hollow sense of bodily self (Corrigan and Christie-Sands, 2020). Relatedly, those with trauma-based dissociative disorders may have adapted to chronic traumatization by suppressing lower-level survival responses and maintaining cortico-cortical hyperconnectivity, resulting in disembodiment and a lack of connection to raw emotion and sensorimotor experience. Indeed, upstream alterations in functional connectivity have been found at the level of the vestibular nuclei (Harricharan et al., 2017), PAG (Harricharan et al., 2016), superior colliculi (Olivé et al., 2018), RAS (Thome et al., 2019), and thalamus (Terpou et al., 2018) reflecting trauma’s widespread, cascading impact on vertical and thus horizontal connectivity in the brain.

### Feeling too much or too little: Over- and under-modulation

**FIGURE 9:**
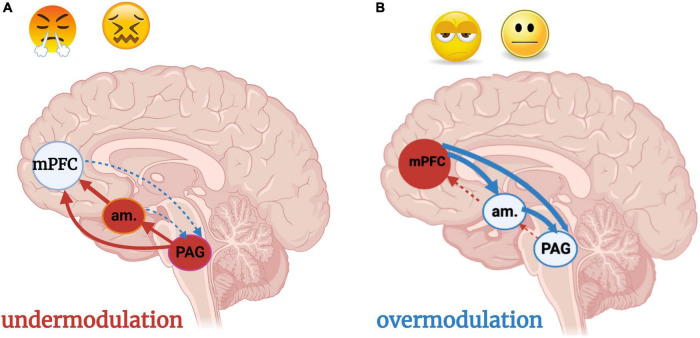
**(A,B)** Under- and over-modulation of emotion and arousal in trauma-related conditions. Image adapted with permission from Nicholson et al. (2017). An individual who encounters a traumatic situation is flooded with negatively valenced sensory stimuli, resulting in hyperactivation of the brainstem and midbrain PAG. An individual who develops a post-traumatic condition experiences persistent over-or under-modulated activity of the PAG and amygdala, resulting in hyper- and hypo- sensory-affective responsivity, respectively, at the level of the PAG. **(A)** Under-modulation or bottom-up predominance (solid arrows) results in hyperarousal and weak top-down neocortical modulation (dashed arrows) of sensory input and emotions. Situations incorporating sensory or emotional stimuli similar to the traumatic event, often over-generalized with regard to valence (i.e., every sensation of butterflies in the stomach is labeled as a sign of danger) triggers hyperarousal and a fight/flight response. **(B)** Over-modulation of lower brainstem and midbrain arousal and alarm centers (amygdala, PAG) is driven by frontal neocortical regions including the mPFC. An individual who experiences chronic, repeated traumatization, such as an adolescent who has grown up in a household with domestic violence and physical abuse, adapts to persistent threat through top-down blunting and avoidance. The adolescent is emotionally numb, withdrawn from relationships, and dissociative due to a scarcity of bottom-up sensory integrative influence and processing. Subcortical regions are prevented from nourishing the cortex with either negative or positive raw affective feelings or sensations unless they are extreme. am, amygdala; mPFC, medial prefrontal cortex; PAG: periaqueductal gray.

A massively hyperactivated brainstem and a tremendous sense of fear lead to prolonged disruptions in physiological homeostasis and exaggerated defensive responses. In PTSD, cortical structures have difficulty modulating incoming sensory and emotional stimuli, contributing to chronic hyper-arousal (Lanius et al., 2010b). Without a grounded, stable, and physiologically regulated *soma* generated through somatic sensory integration, exteroceptive input such as bright lights, sudden noises, and unexpected touch can over-activate the SN, disturb the DMN, and throw multisensory integrative balance off kilter (Figure 9A). Here, one “feels too much” and lacks the ability to self-regulate resulting in aggression, emotional/physical overwhelm, and startle hyperresponsivity. Alternatively, chronic detachment from bodily sensations and emotions in conjunction with fluctuating hypoarousal presents in PTSD + DS. A vicious cycle of overwhelm and shutdown occurs in a desperate attempt to attenuate chronically overbearing feelings. Over-modulation of arousal and affect by prefrontal structures dampens sensory experiences as a protective mechanism, resulting in emotional numbness or feeling “dead inside” (Lanius et al., 2010b) (Figure 9B). Further, chronic childhood traumatization may alter development of SN and DMN circuitry, where highly activating or threatening sensory stimuli feel familiar or normal (Lanius et al., 2020). These individuals may seek to engage the SN and thus gain access to the DMN through intense or primally threatening somatic sensory experiences, such as car racing, extreme sports, and promiscuous sex (van der Kolk, 1989, 2015; Lanius et al., 2020). For severe and lifelong overmodulation, any experience of bodily sensation feels foreign, threatening to flood the cortical defense mechanism created to protect the brain from overwhelm. Those who are overcome by any experience of bodily sensation may avoid certain environments, physical activity, and intimacy which elicit too triggering of sensations. Consideration of pre-reflective sensorimotor brainstem regions in therapeutic attempts to restore, or in the case of chronic childhood trauma, primarily establish, the capacity for safety and trust in the body is crucial.

## The sense of self in health and trauma through a sensory processing lens

The experience of inhabiting one’s own body, or *embodiment*, is an ineffable, subconscious perception rooted in sensorimotor processes (Ciaunica et al., 2021a). Embodiment is a state in which the mind is oriented in physical space, giving rise to a first-person perspective (Lenggenhager et al., 2006, 2007; Blanke and Metzinger, 2009). This spatial union of the mind with the physical body (Ferrè et al., 2014) provides a fundamental reference point for higher-order cortical function as it directs our attention and intentions in volitional thought and action (Panksepp, 1998). Ultimately, it provides us with an anchor to who we are, where we are, and what we want.

### Somatic sensory contributions toward the sense of self

The somatic sensory systems are deemed foundational for the simplest or “minimal” self as it relates to physical world (Panksepp and Northoff, 2009; Metzinger, 2013). The infantile co-embodied self is relational to gravity and interpersonal touch, forming the basis for an agentive, separate self grounded in the physical body (what is happening to “me”- I am moving, I am being touched) (Fotopoulou and Tsakiris, 2017; Crucianelli and Filippetti, 2020; Ciaunica et al., 2021a). Bodily movement, which requires vestibular and somatosensory feedback, begets a sense of a bodily self (Ferrè and Haggard, 2015). This subcortical self-generating system is shared across species and forms the basis for higher forms of selfhood in humans (Panksepp and Northoff, 2009). In corroboration, removing the cortex of young mammals does not appear to compromise the sensorimotor coherence, basic autonomic processes, or raw affective responses required for a brainstem-level sense of self (Kolb and Tees, 1990; Panksepp et al., 1994; Shewmon et al., 1999; Panksepp, 2005; Merker, 2007). Further, manipulations of somatic sensory input in healthy adults have cascading effects on higher self-related constructs such as agency (Synofzik et al., 2008; Lopez et al., 2012; Lenggenhager and Lopez, 2015), embodiment (Schwabe and Blanke, 2008; Ferrè et al., 2014; Macauda et al., 2015; Lopez, 2016; Lopez et al., 2018), self-other distinction and first person perspective (Ferrè et al., 2014), and bodily self-awareness (Lenggenhager et al., 2006, 2012; Blanke, 2012).

**FIGURE 10:**
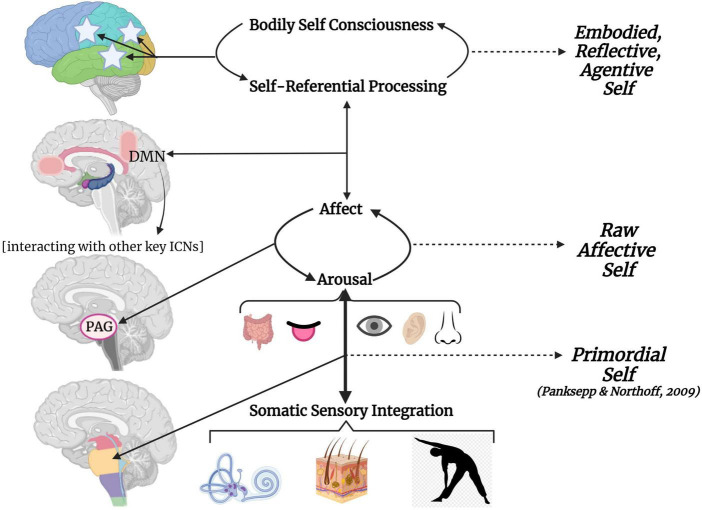
The embodied, reflective, agentive self grounded in somatic sensory integration. This hierarchical model visualizes how somatic sensory processing provides a foundation for exteroceptive and interoceptive sensory processing, influences arousal, mediates how arousal meets affect, and ultimately impacts higher-order self-referential processing and bodily self-consciousness. The left side of the graphic visualizes how neurodevelopmental vertical integration leads to horizontal integration of the cortex; the right side depicts hierarchical complexity of selfhood. Brainstem-level somatic sensory integration provides a subconscious sense of primordial self as it relates to the physical environment. Somatic sensory processing then mediates how arousal meets affect at the level of the midbrain PAG, giving rise to rudimentary awareness of a raw affective self. The PAG as a node of the SN allows for switching between salience detection and self-referential processing within the cortical midline structures (DMN). The DMN fluidly interacts with other key ICNs (SN, CEN) to influence behavioral states. Horizontal integration from cortical midline to lateral structures elaborate upon self-referential processing giving rise to an embodied, reflective, agentive sense of higher self that is anchored to the body. CEN, central executive network; DMN, default mode network; ICN, intrinsic connectivity network; PAG, periaqueductal gray; SN, salience network.

Based on previous work by Panksepp and Northoff (2009) and Ferrè and Haggard (2016), we propose a hierarchical model depicting how somatic sensory information contributes toward primordial selfhood, and how this lays a foundation for higher forms of affective and embodied selves (Figure 10). Sensory integrative processes impact arousal and meet affect at the PAG. The PAG as a node of the SN allows for switching between salience detection and self-referential processing within the cortical midline structures (DMN) (Menon, 2015). These midline structures are involved in self-reflection, autobiographical memory processing and higher-order emotional processing and regulation (Northoff et al., 2006; Tsakiris, 2010). Henceforth, an abstract and subjective self arises from the intricacies of the lateral neocortex given its communication with both more medial and lower reaches of the brain. Without an integrated somatic sensory foundation, abstract knowledge about the body as a physical and emotional agent over which one has control is vulnerable to disruption (Longo et al., 2008, 2009; Ferrè and Haggard, 2015, 2016). Given the functional breakdown of arousal modulation, emotion regulation, sense of self, and SN/DMN connectivity evident in traumatized individuals, we find this model particularly relevant for how somatic sensory processing may contribute toward trauma-related symptomology.

### Trauma and the disembodied self

An ineffable disconnection from a sense of self is extensively reported in PTSD, such as “I am not myself,” and “I feel like an object, not a person” (Foa et al., 1999; Bluhm et al., 2009; Lanius et al., 2011, 2020). Experiences of deep emotion and perceptions of being terrestrially grounded, socially connected, and/or emotionally intuitive are often dulled or completely lost in trauma’s aftermath. Trauma engenders the sense that one’s body is not safe, is not under one’s own control, and/or is a cauldron of disgust and shame- particularly when it is chronic, interpersonal, and early in onset (Frewen and Lanius, 2015). Traumatogenic dissociative symptomology manifests as severe distortions in how the bodily self is perceived and experienced in relation to the outside world and often corresponds with trauma during critical periods of sensorimotor development (van der Hart et al., 2005; van der Kolk, 2005; Schmahl et al., 2010). Without adequate somatic sensory integration, we do not know where our body begins or where it ends; statements such as “I feel as if I am outside my body,” and “I feel like there is no boundary around my body,” highlight how alterations to the bodily self is a common experience in dissociative individuals (Frewen and Lanius, 2015; Lanius et al., 2020; Ciaunica et al., 2021b,c). Severe early life neurodevelopmental disruptions may also give rise to the experience of multiple fragmented, discontinuous, and disembodied selves, such as those experienced in dissociative identity disorder (DID), a condition highly co-existent with early and severe traumatization (Reinders et al., 2003; Dorahy et al., 2014; Schlumpf et al., 2014; Reinders and Veltman, 2021). These various forms of self-disconnection are often impenetrable by means of neocortically targeted therapeutic approaches. A return to the foundation of selfhood via somatic sensory-based therapeutic approaches is crucial to consider.

## What neuroscience can teach us about connecting somatic sensory processing and trauma-related disorders: Treatment implications and future directions

*“While we all want to move beyond trauma, the part of our brain that is devoted to ensuring our survival (deep below our rational brain) is not very good at denial”* (van der Kolk, 2015, p. 2).

If somatic sensory input directly impacts arousal and gives rise to our primordial self, exploiting its power to modulate arousal and bind a coherent sense of self will be an effective therapeutic avenue. Encouraging re-connection with felt bodily experiences of movement and touch within a positively valenced therapeutic alliance will contradict previous negatively valenced multisensory experiences and attachment disruptions (van der Kolk, 2015), leading to upstream regulation of arousal and affect, modulation of exteroceptive sensory input, and embodied cognitive capacities. Although cognitive-behavioral therapies are considered a first line of treatment for traumatized individuals (Malejko et al., 2017; Yang et al., 2018), lower than 50% efficacy in PTSD patients has been reported (Marks et al., 1998; Bradley et al., 2005; Mendes et al., 2008; Kar, 2011), and they can be less beneficial during states of stress (van der Kolk and Fisler, 1995; Raio et al., 2013) or dissociation (Michelson et al., 1998; Rufer et al., 2006; Spitzer et al., 2007; Kleindienst et al., 2011; LeBois et al., 2022; but see also Halvorsen et al., 2014; Zoet et al., 2018; Vancappel et al., 2022). However, we do not suggest a purely “bottom-up” approach; alternatively, we highlight the importance of combining bottom-up with top-down strategies in consideration of brainstem level sensory integration, ontogenetic development, and the fostering of vertical and horizontal integration. The combination of somatic sensory stimulation with awareness, where the individual takes notice of the presence, intensity, and quality of somatic input, may bridge the brain-body disconnect that is often so difficult to address in cognitive therapies alone- thus, somatic sensory approaches may optimize cognitively-focused interventions. Sensory-based interventions are increasing in utility (Fraser et al., 2017; McGreevy and Boland, 2020) and several approaches are discussed in turn.

### Somatic sensory-based psychotherapy

Sensorimotor approaches to psychotherapy, such as Sensorimotor Psychotherapy (SP; Ogden et al., 2006), Somatic Experiencing (Levine, 2010), and Sensory Motor Arousal Regulation Therapy (SMART; Warner et al., 2013), emphasize mindful attention to touch, postures, and movement. Grounded in concepts of experience-dependent neuroplasticity and Hebbian learning (Hebb, 1949; Rao and Sejnowski, 2001), these approaches are posited to repeatedly evoke an organizing response conducive to environmental and self-mastery, or the consistent and satisfying ability to produce meaningful goal-directed action (Ayres, 1972). Small-scale clinical trials of SP-informed group therapy (Langmuir et al., 2012; Gene-Cos et al., 2016; Classen et al., 2021) and case-studies of SMART (Warner et al., 2014; Finn et al., 2018) and SE (Payne et al., 2015) have shown promising results in arousal regulation and reductions in PTSD symptoms. Deep brain re-orienting (DBR) is an approach that similarly targets the multisensory integrative brainstem and midbrain regions by specifically focusing on orienting and postural responses. While trauma results in overwhelming alarm (at the level of the LC and SC) and/or affect (at the level of the PAG), therapeutic attention to the muscular tension in the neck, indicative of SC activation (Corneil and Camp, 2018) elicited in response to a traumatic memory provides information on its pre-affective components. DBR is highly relevant for those with chronic early life trauma given the importance of muscle activation, head turning, and reaching in both approach toward and withdrawal from early attachment figures (Corrigan and Christie-Sands, 2020). These approaches exploit subcortical processes by means of somatic sensory feedback and facilitate awareness and experience of somatic sensations in the present moment of safety as opposed to a past trauma (Fisher, 2011, 2019). This process may be the antithesis to dissociative flashbacks, derealization, and depersonalization and a catalyst for reinstating a sense of agency and trust in the body.

### Eye movement desensitization and reprocessing

Eye Movement Desensitization and Reprocessing (EMDR) therapy utilizes saccadic eye movements to elicit an innate relaxation response and integrate maladaptively stored trauma memories (Shapiro, 1995; Oren and Solomon, 2012). Although the mechanism behind EMDR’s therapeutic benefits remains unclear, oculomotor muscles have extensive reciprocal connections with the brainstem reticular formation, vestibular nuclei, and superior colliculi (Horn and Adamzyk, 2012; Harricharan et al., 2019) influencing arousal and multisensory integration. Reduced physiological arousal is reported during EMDR sessions (Aubert-Khalfa et al., 2008; Sack et al., 2008; Söndergaard and Elofsson, 2008), which then attenuates the intensity of negative affect (Oren and Solomon, 2012). Significant reductions in PTSD symptoms have been found in as few as three to eight sessions (Rothbaum, 1997; Wilson et al., 1997; Marcus et al., 2004) although longer durations are recommended for severe and chronic early life traumatization (van der Kolk et al., 2007).

### Neurofeedback

Electroencephalographic (EEG) neurofeedback (NFB) training is a non-invasive technique based on the premise that cognitive and motor function depend upon proper communication of neuronal assemblies organized into constellatory networks (Neuper and Pfurtscheller, 2001; Nicholson et al., 2020b). The provision of sensory feedback reflecting neuronal activity in real time has shown to be an effective way to voluntarily promote low frequency, high amplitude alpha oscillations associated with a calm, introspective state. Alpha synchrony may act as a sensory gating mechanism to inhibit irrelevant stimuli (Herrmann and Knight, 2001; Jensen and Mazaheri, 2010; Jones and Sliva, 2020; Saj et al., 2021) and is typically found in deeper midline structures, including the thalamus (Lopes da Silva, 1991) supporting its role in vertical integration. Alpha-targeted neurofeedback has been shown to normalize SN and DMN connectivity and diminish symptom severity in PTSD (Kluetsch et al., 2014; Nicholson et al., 2016, 2020a; van der Kolk et al., 2016; Ros et al., 2017; Rogel et al., 2020) and relieve sensory hyper-sensitivities (Hamed et al., 2022).

### Play therapy

For the traumatized child, play-based approaches inclusive of vestibular and somatosensory feedback and affective interpersonal dynamics invite the clinician into the child’s primordial relational world. The richness of vestibular and somatosensory input in classical childhood games spanning generational and cultural boundaries such as “peek-a-boo,” “hide-and-seek,” and “tag” informs the importance of somatic sensory systems in neurodevelopment and in navigating social relationships. For the traumatized child, engaging in these games within a therapeutic context has the promise of re-organizing brainstem level sensory and affective circuitry. By further exploiting the power of a basic affective motivational (PLAY) system at the level of the PAG (Panksepp, 1998), movement play-based therapy restores the intrinsic capacity for orienting to others, enriches the child’s capacity for positive affective responding, and restores the primal urge to seek relational connection through somatic sensory experience (Crenshaw and Kenney-Noziska, 2014; Corrigan and Christie-Sands, 2020).

### Yoga

The importance of mindful movement and proprioceptive feedback to ground the body and attune to its needs has been implicit in yogic tradition for centuries. Trauma-informed yoga practitioners guide clients in tolerating titrated somatic sensation and/or the affective states somatic sensory input can arouse. Additionally, mindful movements and postures support top-down regulation of somatically induced arousal. Trauma-Sensitive Yoga (TSY; Emerson et al., 2009; Emerson and Hopper, 2011) is one such example of the utilization of trauma-informed yoga within a therapeutic group setting. Two randomized controlled trials of women with treatment-resistant PTSD showed reductions in symptoms and dissociation; the shorter-duration study (10-weeks) resulted in symptom decreases comparable to psychopharmaceutical and psychotherapeutic interventions (van der Kolk et al., 2014) while extended yoga practice (20-weeks) drove even greater improvements (Price et al., 2017).

### Equine assisted therapy

For individuals with severe early life trauma and attachment disruptions, feeling safe within any human relationship may be difficult or impossible. Equine-facilitated psychotherapy (EFP) facilitates attunement to horses (Lentini and Knox, 2015), where individuals learn how to read non-verbal cues and be within a non-threatening, attuned, co-regulatory relationship. EFP involves grooming and caring for the horse to build trust through somatosensory means, as well as mounting (riding the horse with or without a saddle, maintaining postural control, riding with eyes closed) with the guidance of a trained therapist (Naste et al., 2018). A sense of mastery is built through client-directed touch, balance, and bodily control within the context of a safe, co-regulatory dynamic (Kendall and Maujean, 2015). Several non-randomized, small sample-sized studies reported reductions in depressive and PTSD-associated symptoms after EFP (Nevins et al., 2013; Signal et al., 2013; Yorke et al., 2013; Kemp et al., 2014; McCullough et al., 2015; for a review, see Lentini and Knox, 2015; O’Haire et al., 2015).

### Expressive arts therapy

Expressive arts therapy, including the therapeutic application of music, dance, theatre, art, and creative writing, is another approach that consistency engages the body through purposeful action (Malchiodi, 2020). Here, the “felt” sense of trauma, experienced within and through the body, is expressed in non-verbal, sensory-based, action-oriented artforms that tap the implicit embodied experiences of trauma defying narrative or logical expression (Terr, 1990; Malchiodi, 2020). Each form of expressive arts therapy incorporates the physical body and meaningful, purposeful action which produces an intentional result, forming or re-forming sensory-motor feedback loops which engender a sense of agency, power, and positive self-environment relationship. Further, the non-verbal rhythmicity inherent in music therapy may provide vibratory stimulation and an external source of rhythm when internal rhythms and sensorimotor synchrony is disrupted due to somatic sensory disintegration. Moderately reduced trauma-related symptoms have been reported in individuals receiving art therapy (Chapman et al., 2001; Curry and Kasser, 2005; Henderson et al., 2007; for a review, see Schouten et al., 2015) and music therapy (Carr et al., 2012) interventions for PTSD.

## Conclusion

Janet (1889) classically characterized trauma as an inability to be fully alive in the present moment. If the essence of trauma is the severance of affiliative bonds with the self and others (van der Kolk, 1987), and healing from trauma is indicated by one’s ability to be in an optimal state of arousal while experiencing affective sensations/emotions in the body, then further research into the neurobiological correlates of vestibular and somatosensory processing in trauma-related disorders is imperative and pressing. For those flooded by sensation and emotion, building tolerance of somatic sensation using top-down strategies is essential. For those wired to “shut down” or avoid somatic sensation as a defense mechanism, identification of somatic cues related to shifts in arousal and/or emotion may be facilitated through titrated somatic sensory feedback. Therapeutic approaches centered around attunement to somatic sensory-affective experiences may approach the roots of the dynamic system. This may in turn facilitate a mind-body connection that was, or was not ever, there before, thereby laying the foundation for the restoration of an embodied self that is capable of feeling fully alive in the aftermath of trauma.

## Author contributions

Both authors listed have made a substantial, direct, and intellectual contribution to the work, and approved it for publication.
